# Pachypodol attenuates Perfluorooctane sulphonate-induced testicular damage by reducing oxidative stress

**DOI:** 10.1016/j.sjbs.2021.12.012

**Published:** 2021-12-11

**Authors:** Muhammad Umar Ijaz, Ayesha Rauf, Shama Mustafa, Hussain Ahmed, Asma Ashraf, Khalid Al-Ghanim, Satyanarayana Swamy Mruthinti, S. Mahboob

**Affiliations:** a Department of Zoology, Wildlife and Fisheries, University of Agriculture, Faisalabad, Pakistan; b Department of Zoology, The University of Buner, Khyber Pakhtunkhwa, Pakistan; c Department of Zoology, Government College University, Faisalabad, Pakistan; d Department of Zoology, College of Science, King Saud University, Saudi Arabia; e Department of Biology, University of West Georgia, Carrollton, GA, USA

**Keywords:** Perfluorooctane sulfonate, Pachypodol, Testicular toxicity, Reactive oxygen species, Oxidative stress, Antioxidant

## Abstract

Perfluorooctane sulfonate (PFOS) is an endocrine disruptor chemical (EDC) with potentially adverse effects on the male reproductive system. Pachypodol (5,4′-dihydroxy-3,7,3′-trimethoxyflavone) is a promising flavonoid isolated from Pogostemon cablin (Blanco) Benth that shows a broad range of pharmacological properties. However, the potential curative effects of pachypodol on testicular toxicity are not available until now. Therefore, this research was proposed to examine the efficiency of pachypodol against PFOS-induced testicular toxicity in adult male rats. The experiments were conducted on Sprague-Dawley rats (n = 48), which were equally distributed into four groups: control, PFOS (20 mg/kg), PFOS + Pachypodol (20 mg/kg + 10 mg/kg respectively), and Pachypodol (10 mg/kg). After 56 days of treatment, testes were excised by slaughtering rats, weighed, and stored till further analysis. The estimated parameters include biochemical markers, spermatogenic indices, hormonal and histopathological profiles. PFOS exposure disturbed the biochemical profile by altering the antioxidant/oxidant balance. For instance, it decreased the activities of catalase (CAT), superoxide dismutase (SOD), glutathione peroxidase (GPx), and glutathione reductase (GSR) while increasing the concentration of reactive oxygen species (ROS) and level of thiobarbituric acid reactive substances (TBARS). PFOS intoxication also led to a notable decline in viability, motility, epididymal sperm count, and the number of HOS coiled-tail sperms, whereas the higher level of abnormality in the head, mid-piece, and tail of sperms were observed. Besides, it lowered luteinizing hormone (LH), follicle-stimulating hormone (FSH), and plasma testosterone. In addition, PFOS exposure led to histopathological damages in testicles. However, pachypodol treatment potently alleviated all the illustrated impairments in testes. Conclusively, our results demonstrate the promising free-radical scavenging activity of pachypodol, a novel phytochemical, against the PFOS-instigated testicular dysfunctions.

## 1 Introduction

Per-and polyfluoroalkyl substances (PFASs) are synthetic chemicals that show adverse effects on the ecosystem and human health (Zhang et al., 2020). According to some global statistics, over 4700 PFASs have been identified (OECD, 2018). Among these PFASs, perfluorooctane sulfonate (PFOS) is a widely used surfactant due to its excellent thermal stability and hydrophobic properties, which adversely affect humans and wildlife (Chen et al., 2021). Environmental sources of PFOS exposure include commercial and industrial products, such as cookware, furniture, household cleaners, clothing, and firefighting foam (Sunderland et al., 2019). Furthermore, drinking water and contaminated foods are the chief sources of PFOS exposure to humans (Alam et al., 2021). Primary routes of PFOS exposure to the human body are ingestion, inhalation, and dermal contact (Poothong et al., 2020). Inside the body, PFOS bind to serum proteins and renal transporter proteins that restrict its discharge in urine due to its reabsorption in the kidneys (Worley and Fisher, 2015). The estimated half-life of PFOS in the human body is 3.4 years (Li et al., 2018).

It is evident from multiple kinds of researches, PFOS exposure in model animals potently influences various biological processes, i.e., neurotoxicity (Negri et al., 2017), endocrine disruption (Coperchini et al., 2017), blood-testis barrier impairment (Wan et al., 2014), and developmental toxicity in the reproductive system (Xu et al., 2017). Furthermore, PFOS prevents sex maturation by acting as an antiandrogen (Di Nisio et al., 2019). The mediator of PFOS-induced reproductive toxicity is oxidative stress (OS) (Oseguera-López et al., 2020). Previous literature shows that OS deteriorates spermatozoa, leading to male infertility (Agarwal and Sengupta, 2020). It arises when the overproduction of ROS disrupts the oxidant/antioxidant balance (Yilmaz et al. (2017)). As feedback, this redox imbalance leads to lipid peroxidation (LP) in the testicle and sperm cells as testes hold large amounts of polyunsaturated fatty acids (PUFA) (Bisht et al., 2017). Hence, OS-induced LP can be deleterious to steroidogenesis and spermatogenesis in testicular tissues (Ijaz et al., 2021a). PFOS exposure in humans can cause reduced sperm motility (Song et al., 2018), epididymal sperm count (Gao et al., 2017), and deterioration of semen quality and quantity (Toft et al., 2012). Thus, after analyzing the various sources of PFOS exposure and their adverse effects on human health, particularly on testes, research on remedies against PFOS-instigated toxicities is desired.

Flavonoids are a polyphenolic family of plants' bioactive secondary metabolites (Nabavi et al., 2020). The chief flavonoid reservoirs are vegetables and fruits (Liu et al., 2018). Among these plant polyphenolic families, pachypodol (4′,5-dihydroxy-3,3′,7-trimethoxyflavone) is a natural dietary flavonoid, which is isolated from Pogostemon cablin (Blanco) Benth with reported antioxidant (Kim et al., 2019), anti-apoptotic (Zhang et al., 2021), antimicrobial (Krithika et al., 2021), and cytoprotective (Kim et al., 2019) properties. Despite these potential curative effects of pachypodol, its ameliorative potential on testicular toxicity is not available until now. Therefore, current research ascertained the alleviated potential of pachypodol for PFOS-instigated testicular toxicities. Hence, to accomplish this aim, PFOS and pachypodol were orally cotreated to adult male Sprague-Dawley rats for 56 days, after which OS and resultant pathological alterations, such as sperm count, motility, morphology, hormone levels, and histopathology of testicular tissues were analyzed.

## 2 Materials and methods

### 2.1 Chemicals

PFOS and Pachypodol were purchased from Sigma-Aldrich, Germany.

### 2.2 Animals

Adult male Sprague-Dawley rats (180 ± 20 g) were obtained from the animal house of the University of Agriculture Faisalabad (UAF), which were kept in steel cages at standard 12 h light/dark cycle, temperature (22–25 °C), and humidity (45 ± 5%). Food and Tap water (H_2_O) were given *ad libitum*. This study was conducted in compliance with the guidelines for the supervision and handling of animals stated by the institutional ethics committee, University of Agriculture, Faisalabad.

### 2.3 Experimental design

Rats (n = 48) were randomly divided into four groups (12 rats/group): Control, PFOS, PFOS + Pachypodol, and Pachypodol alone. 20 mg/kg dose of PFOS and 10 mg/kg dose of Pachypodol were used in this experiment, and all the doses were given orally. Rats were handled according to the European Union of animal care and experimentation (CEE Council 86/609) guidelines. At the end of 56 days of treatment, rats were killed by decapitation, and testes were excised, weighed, and stored at −80 °C till further analysis.

### 2.4 Biochemical markers

The activities of catalase (CAT), superoxide dismutase (SOD), glutathione peroxidase (GPx), and glutathione reductase (GSR) were quantified by following the previous techniques (Chance and Maehly, 1955, Kakkar et al., 1984, Lawrence and Burk, 1976); Carlberg and Mannervik, 1975), whereas the concentration of reactive oxygen species (ROS), as well as thiobarbituric acid reactive substances (TBARS) level, was measured according to the protocols of Hayashi et al., 2007, Iqbal et al., 1996 respectively.

### 2.5 Semen analysis

Caudal piece of epididymis was isolated to take the semen samples. Initially, the epididymal part was minced in 5 mL of physiological-saline and then heated for a half-hour at 37 °C to let the sperms abscond from the epididymis. Sperm motility was recorded with the help of a phase-contrast microscope at 400X (Kenjale et al., 2008). Sperm viability was estimated by eosin and nigrosin staining, followed by microscopic evaluation. Moreover, a hemocytometer was employed to count epididymal sperm (Yokoi et al., 2003). Furthermore, morphological anomalies of head, tail, and mid-piece of sperm were ascertained by the process of Cao et al. (2017).

### 2.6 Hypo-osmotic swelling (HOS) test

The HOS test was employed to measure the morphological sperm plasma membrane integrity. In the first step, 20 μL of semen sample was placed in 180 μL of fructose solution at 80 mOsm/L osmotic pressure for about 20 min. After subsequent incubation and mixing, the sperm were stained with eosin and nigrosin. Lastly, microscopically (400X), 200 spermatozoa with swollen and non-swollen tails were analyzed (Correa and Zavos, 1994).

### 2.7 Hormonal assay

Specific enzyme-linked immunosorbent assay (ELISA) kits were employed to quantify the serum levels of luteinizing hormone (LH), follicle-stimulating hormone (FSH), and plasma testosterone.

### 2.8 Histopathology

Tissue samples for histopathological analysis were obtained from the left testis of the rats. For light microscopy, samples were fixed for about 48 h in a 10% formaldehyde solution, then immediately dehydrated in rising grades of alcohol and encased in paraffin wax. Later these paraffin-inserted blocks (5 μm) were cut and stained with hematoxylin-eosin (H & E). Lastly, Leica LB microscope and Image-J2X software were used respectively to take or analyze the images of the sample.

### 2.9 Statistical analysis

Data are shown as means ± SEM. The normality of data was first tested using Levene’s test. A one-way analysis made comparisons of the differences of variance (ANOVA) followed by Fisher's LSD for multiple comparisons or nonparametric Kruskal-Wallis as appropriate. Statistical significance was taken at P < 0.05. All analyses were performed using Minitab software.

## 3 Results

### 3.1 Effect of pachypodol on biochemical markers

Table 1 represents the mean values of biochemical markers. PFOS exposure substantially (p < 0.05) reduced the activity of enzymatic antioxidants such as CAT, SOD, GPx, or GSR, while augmenting the ROS and TBARS level concentration in PFOS-treated rats in comparison to the control rats. Co-administration of pachypodol with PFOS provoked substantial (p < 0.05) elevation in CAT, SOD, GPx, and GSR activities along with a significant (p < 0.05) decrease in the concentration of ROS and TBARS levels in contrast to the PFOS-exposed rats. Furthermore, the pachypodol alone treatment group did not exhibit any significant (p < 0.05) variations in the status of the biochemical assay as compared with the control group.

**Table 1:** Mean ± SEM of biochemical markers in the testicles of control, PFOS-treated, cotreated, and pachypodol groups.

**Parameters**	**Groups**			
	Control	PFOS	PFOS + Pachypodol	Pachypodol
**CAT** (U/mg protein)	9.17 ± 0.23^a^	5.38 ± 0.15^b^	8.28 ± 0.11^a^	9.24 ± 0.25^a^
**SOD** (U/mg protein)	6.55 ± 0.35^a^	3.22 ± 0.06^b^	5.88 ± 0.22^c^	6.58 ± 0.41^a^
**GPx** (U/mg protein))	18.32 ± 0.59^a^	9.17 ± 0.17^b^	14.72 ± 0.41^c^	18.55 ± 0.69^a^
**GSR** (nm NADPH oxidized/min/mg tissue	5.07 ± 0.15^a^	1.41 ± 0.19^b^	3.84 ± 0.100^c^	5.13 ± 0.17^a^
**ROS** (U/mg tissue)	1.06 ± 0.16^a^	7.11 ± 0.27^b^	2.02 ± 0.100^c^	1.03 ± 0.13^a^
**TBARS** (nM/min/mg tissue)	12.25 ± 1.15^a^	28.43 ± 0.74^b^	16.33 ± 0.66^c^	12.14 ± 1.12^a^

Values having various superscripts are considerably (p < 0.05) distinct from other groups.

### 3.2 Effect of pachypodol on semen analysis

Table 2 shows the semen indices. PFOS exposure substantially (p < 0.05) reduced the sperm viability, motility, epididymal sperm, and HOS coil-tailed sperm count while increasing the morphological sperm anomalies (head, mid-piece, and tail) in contrast to the control group. However, pachypodol-supplementation substantially (p < 0.05) inverted all these sperm indices to a normal state in the co-treated group compared to the PFOS-induced group. Nevertheless, only pachypodol-administered rats exhibited a regular semen profile as in the control rats.

**Table 2:** Mean ± SEM of semen assay in control, PFOS-treated, cotreated, and pachypodol groups.

**Parameters**	**Groups**			
	Control	PFOS	PFOS + Pachypodol	Pachypodol
**Motility** (%)	88.88 ± 3.47^a^	35.70 ± 1.11^b^	71.58 ± 1.98^c^	89.97 ± 3.85^a^
**Dead Sperms** (%)	7.80 ± 1.35^a^	86.56 ± 1.79^b^	12.22 ± 4.54^c^	7.41 ± 1.29^a^
**Head Abnormality** (U/mg protein)	4.85 ± 0.50^a^	19.45 ± 1.25^b^	8.59 ± 0.73^c^	4.64 ± 0.44^a^
**Mid Sperm Abnormality** (%)	0.70 ± 0.09^a^	8.91 ± 0.55^b^	1.65 ± 0.13^c^	0.67 ± 0.15^a^
**Tail Abnormality** (%)	1.51 ± 0.08^a^	15.48 ± 0.63^b^	3.52 ± 0.22^c^	1.45 ± 0.12^a^
**Hypo-osmotic swelled sperm count (HOS)** (%)	86.17 ± 1.91^a^	25.57 ± 1.85^b^	65.46 ± 1.67^c^	86.62 ± 2.08^a^
**Epididymal Sperm Count** (million/mL)	29.20 ± 1.31^a^	11.69 ± 0.78^b^	24.68 ± 0.65^c^	29.25 ± 1.77^a^

Values having various superscripts are considerably (p **<** 0.05) distinct from other groups.

### 3.3 Effect of pachypodol on hormonal assay

Table 3 demonstrates the mean values of the hormonal assay. PFOS intoxication substantially (p < 0.05) reduced the LH, FSH, and plasma testosterone levels in the PFOS group compared to the control group. However, cotreatment of pachypodol with PFOS significantly (p < 0.05) restored the above-stated hormonal levels compared to the PFOS group. Besides, the pachypodol-treated group displayed the average level of the hormonal assay as shown in the control group.

**Table 3:** Mean ± SEM of the hormonal assay in control, PFOS-treated, cotreated, and pachypodol groups.

**Parameters**	**Groups**			
	Control	PFOS	PFOS + Pachypodol	Pachypodol
**LH (**ng/ml)	2.35 ± 0.11^a^	0.82 ± 0.13^b^	2.05 ± 0.07^a^	2.39 ± 0.13^a^
**FSH (**ng/ml)	4.06 ± 0.12^a^	1.29 ± 0.10^b^	3.72 ± 0.19^a^	4.11 ± 0.13^a^
**Plasma testosterone** (ng/ml)	4.61 ± 0.09^a^	2.10 ± 0.08^b^	3.73 ± 0.07^a^	4.68 ± 0.11^a^

Values having various superscripts are considerably (p **<** 0.05) distinct from other groups.

### 3.4 Effect of pachypodol on histopathology

**Fig. 1:**
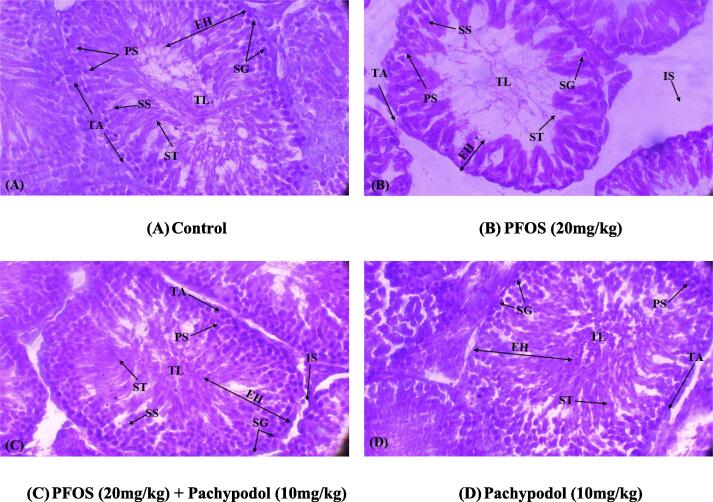
(A) Control group demonstrating thick germinal epithelium including different stages of germ cells and the slender luminal area carrying spermatozoa; (B) PFOS group displaying sloughing of the epithelial layer, vacant lumen, and degeneration of IS; (C) PFOS + Pachypodol group displaying reduced sloughing of germinal epithelium, TL filled with ST and degenerated IS; (D) Pachypodol group representing compact ST with less IS. IS Interstitial spaces; TL: Tubular lumen; EH: Seminiferous Epithelial height; ST: Seminiferous tubules; TA: Tunica albuginea; SG: Spermatogonia; ST: Spermatids; PS: Primary spermatocytes; SS: Secondary spermatocytes.

The histopathological alterations following the PFOS and pachypodol administration are shown in Table 4 and Fig. 1. PFOS induction substantially (*p* < 0.05) lessened the diameter, epithelial height of seminiferous tubules, and the thickness of tunica albuginea. Furthermore, it scaled up the luminal diameter of tubules. PFOS-intoxication also significantly (*p* < 0.05) reduced germ cell count such as spermatogonia, spermatids, primary and secondary spermatocytes compared with the control group. Nonetheless, pachypodol supplementation in the cotreated group substantially (*p* < 0.05) recovered all these structural anomalies and the number of germ cells in testicles compared to the PFOS-induced group. However, there were no significant (*p* < 0.05) differences among the mean values of pachypodol-treated and the control groups.

**Table 4:** Mean ± SEM of histopathology of rat testicles in control, PFOS-treated, cotreated, and pachypodol groups.

**Parameters units**	**Groups**			
	Control	PFOS	PFOS + Pachypodol	Pachypodol
**Interstitial Spaces** (µm**)**	9.84 ± 0.41^a^	36.72 ± 3.35^b^	15.98 ± 0.75^c^	9.54 ± 0.35^a^
**Tunica Albuginea** (µm**)**	68.76 ± 2.07^a^	15.26 ± 0.73^b^	53.55 ± 1.34^c^	70.53 ± 2.85^a^
**Seminiferous Tubules** (µm**)**	356.26 ± 7.01^a^	110.63 ± 9.78^b^	306.15 ± 4.45^c^	348.73 ± 7.69^a^
**Seminiferous Tubule Epithelial Height** (µm**)**	94.08 ± 3.14^a^	31.71 ± 1.60^b^	74.56 ± 2.28^c^	93.00 ± 4.44^a^
**Tubular Lumen** (µm**)**	24.79 ± 1.21^a^	84.18 ± 1.63^b^	37.8 ± 2.00^a^	25.06 ± 1.66^a^
**Spermatogonia**	61.43 ± 1.61^a^	19.12 ± 1.41^b^	52.96 ± 1.64^a^	62.55 ± 4.48^a^
**Spermatids**	48.99 ± 2.00^a^	22.73 ± 0.99^b^	44.36 ± 0.83^c^	49.54 ± 2.05^a^
**Primary Spermatocytes**	45.09 ± 1.06^a^	18.29 ± 1.00^b^	38.21 ± 0.87^c^	45.40 ± 1.38^a^
**Secondary Spermatocytes**	34.01 ± 0.75^a^	11.74 ± 0.60^b^	28.65 ± 0.59^c^	34.31 ± 0.82^a^

Values having various superscripts are considerably (p **<** 0.05) distinct from other groups.

## 4 Discussion

Due to wide occurrence, resistance to degradation, or bioaccumulation in the environment, PFOS is considered one of the emerging persistent organic pollutants (POPs) and exerted potentially deleterious impacts on the ecosystem and humans, including their male reproductive system (Tescari and Nisio, 2017). Administration of this drug causes excessive ROS generation, which results in an imbalance of antioxidant and pro-oxidant enzymes (Li et al., 2017). Furthermore, the over-generation of ROS is one of the main reasons behind the deteriorated semen quality (Ijaz et al., 2020). Thus, the use of antioxidants potently alleviates the PFOS-instigated oxidative damage in testicular tissues. Therefore, in the current investigation, pachypodol, a potent antioxidant flavonoid, was used to cure PFOS-generated testicular dysfunctions in rats.

As evident, ROS are free radicals that arise from oxygen through orderly cellular metabolism (Staveness et al., 2016). Nitric oxide (NO), hydrogen peroxide (H_2_O_2_), hydroxyl radical (OH), and superoxide anion (O_2_^–^) are the central reactive oxygen and nitrogen species (Lü et al., 2010). OS occurs when there is instability between ROS concentration and the body's antioxidant defense system (Pisoschi et al., 2021). Antioxidant defense systems include CAT, SOD, GPx, and GSR that act as the major lines of the body's defense and protect biomolecules (lipids, protein, deoxyribose nucleic acid; DNA) from OS by decreasing the overproduction of ROS (Papas et al., 2019). SOD is a vital free radical scavenger enzyme that converts O_2_^–^ into H_2_O_2_ (Bromfield, 2016), then CAT transforms H_2_O_2_ into H_2_O (Jonakova et al., 2010). GPx helps in the degradation of H_2_O_2_ by converting reduced glutathione (GSH) into glutathione disulfide (GSSG) (Schjenken and Robertson, 2014). On the other hand, GSH functions as an electron donor in these reactions. The concentration of GSH is retained by GSR, which is crucial for sperm production (Ali et al., 2020). Thus, a decline in the activities of antioxidant enzymes elevates the concentration of ROS, which attacks the PUFA in the sperm plasma membrane and triggers a cascade of chemical reactions, which is known as LP (Fois et al., 2018). In this study, TBARS as a marker of LP increased the membrane permeability. As confirmed by the outcomes of the current study, PFOS exposure decreased the activities of cellular enzymatic defense such as CAT, SOD, GPx, and GSR, while increasing the concentration of ROS and TBARS levels. However, this reduced activity of antioxidant enzymes in rat testicles was enhanced by the cotreatment of rats with pachypodol, probably due to its antioxidant property.

Secondly, PFOS-intoxication caused a decrease in viability, motility, epididymal sperm, and HOS coiled-tail sperm count in addition to higher levels of abnormality in the head, mid-piece, and tail of sperm. OS plays a significant role in testicular impairments (Ijaz et al., 2021b). The imbalance of the antioxidants/oxidants or ROS-prompts membrane damage due to the high level of PUFAs in sperm might be the reason behind the distorted integrity of sperm, as confirmed by the HOS test in the present study. Nevertheless, pachypodol administration successfully resettled all the spermatogenic damages due to its potent ROS scavenging activity.

Moreover, the successful regulation of the reproductive process depends upon the hormones working in the hypothalamic-pituitary-gonadal (HPG) axis (Acevedo-Rodriguez et al., 2018). In males, it affects the subsistence of testosterone generation and spermatogenesis, which is critically reliant on the two pituitary gonadotropins, LH and FSH (Sengupta et al., 2019). LH accounts for the stimulation of Leydig cells (LCs) to produce testosterone, whereas FSH sustains the proliferation of immature Sertoli cells (SCs) in synergy with testosterone (androgen) (Ramaswamy and Weinbauer, 2014. The generation of nutrients and molecules is required for spermatids' growth and the release of sperm (Oduwole et al., 2018). In the contemporary study, PFOS exposure lessened the gonadotropins and androgen levels, which may be due to a disruption of the normal physiological axis connecting the hypothalamus with the pituitary gland and the testes. However, pachypodol treatment potentially ameliorated PFOS-induced dysregulation of the hormonal assay.

Similarly, spermatogenesis is a crucial phase in the male reproductive system. During spermatogenesis, germ cells in the seminiferous tubules go through mitotic and meiotic division, forming haploid spermatids. These spermatids, later on, remodel and finally develop into mature spermatozoa (sperm) (Nishimura and L’Hernault, 2017). The germ cells manifest androgen receptors and thus are steadily react to the hormonal milieu enclosing them. Any alterations in the hormone levels could appear in damaged spermatogenesis (Griswold, 2016). Outcomes of the current research demonstrate that PFOS exposure caused severe histopathological alterations in the testis of the rats. In the current study, the decline in the hormonal levels after PFOS exposure is the probable reason behind the spermatogenic impairments, decreased seminiferous epithelial height, tubular diameter, and tunica albuginea thickness; besides escalated interstitial space and diameter of the tubular lumen. However, pachypodol treatment significantly alleviated the histopathological impairments provoked by PFOS. Pachypodol improved histopathological profile might be due to its antioxidant, androgenic, and anti-apoptotic potential.

## 5 Conclusion

In conclusion, exposure to PFOS prompted damages in semen, hormonal, and histopathological profiles in adult male Sprague-Dawley rats. Additionally, the activities of antioxidant enzymes, the concentration of ROS, and the TBARS level presented a state of imbalance, thereby impairing the structure and physiology of the entire male reproductive system. Nonetheless, pachypodol treatment remarkably mitigated PFOS-induced impairments in all the above-stated parameters due to its antioxidant androgenic potential. Depending on these in vivo findings, further researches on the remedial impacts of pachypodol, particularly on the reproductive system, are needed to entirely explore its health-promoting potential that forms the scientific foundation for supporting its consumption.

## Declaration of Competing Interest

The authors declare that they have no known competing financial interests or personal relationships that could have appeared to influence the work reported in this paper.
